# Leisure time physical activity: a protective factor against metabolic syndrome development

**DOI:** 10.1186/s12889-023-17340-w

**Published:** 2023-12-07

**Authors:** Myong-Won Seo, Youngseob Eum, Hyun Chul Jung

**Affiliations:** 1 Departments of Exercise Science, David B. Falk College of Sport and Human Dynamics, Syracuse University, Syracuse, NY USA; 2 Department of Geography and Earth Sciences, The University of North Carolina at Charlotte, Charlotte, NC USA; 3 Sports Science Research Center, College of Physical Education, Kyung Hee University, Yongin-si, Gyeonggi-do Republic of Korea; 4 Department of Sports Coaching, College of Physical Education, Kyung Hee University, Yoinin-si, Gyeonggi-do Republic of Korea

**Keywords:** Leisure-time physical activity, Occupation physical activity, Cardiometabolic abnormalities, Does-response relationship

## Abstract

Physical activity (PA) is a modifiable factor in preventing/treating cardiometabolic disease. However, no studies have yet compared specific moderate-to-vigorous PA (MVPA) domains with the risk of metabolic syndrome (MetS) in detail. Here, the present study was conducted to examine the impact of different MVPA domains (leisure-time PA (LTPA) vs. occupational PA (OPA) vs. total MVPA) on the risk of MetS in Korean adults. Materials and methods: Data from the 2014 to 2021 Korea National Health and Nutrition Examination Survey were analyzed (N = 31,558). MetS was defined according to the criteria by revised NCEP/ATP-III. The domain-specific MVPA was assessed using the K-GPAQ. The LTPA and OPA status were classified into four categories: (1) 0 min/week, (2) 1 to 149 min/week, (3) 150 to 299 min/week, and 4) ≥ 300 min/week. In addition, the present study calculated total MVPA as a sum of OPA and LTPA and further classified it into six groups; (1) 0 min/week, (2) 1 to 149 min/week, (3) 150 to 299 min/week, (4) 300 to 449 min/week, (5) 450 to 599 min/week, 6) ≥ 600 min/week. Results: The ≥ 300 min/week and the 150 to 299 min/week of LTPA showed better outcomes in cardiometabolic disease risk factors and surrogate markers of insulin resistance compared with the 0 min/week of LTPA regardless of adiposity status. Risk of MetS in ≥ 300 min/week of LTPA was lower than in 0 min/week, 1 to 149 min/week, and 150 to 299. In addition, LTPA was significantly associated with a risk of the MetS in a curvilinear dose-response curve, however, no significant effects of a non-linear relationship between OPA and risk of the MetS. Conclusions: Our findings showed that LTPA was associated with a risk of MetS with a dose-response curve, whereas no significant non-linear effects were found between OPA and the risk of MetS. Therefore, the MVPA domain is an independent factor of the risk of MetS.

## Introduction

Physical activity (PA) has a strong impact on overall health conditions such as morbidity and mortality risk [1]. The PA guideline recommends that adult individuals are required to engage in a minimum of 150 min of moderate-intensity activity per week or vigorous-intensity activity for at least 75 min per week, or an equivalent combination of moderate to vigorous intensity PA (MVPA), with 1 bout of at least 10 min duration to maintain overall health and quality of life [2]. However, in a pooled analysis of 358 survey factors across 168 countries with 1·9 million populations, the global prevalence of physical inactivity is estimated to be 27.5% (95% CI; 25.0-32.2) in adults [3].

To date, World Health Organization (WHO) has suggested a Global Action Plan on Physical Activity 2018–2030 (GAPPA) [4], which aims to increase PA levels and achieve the target of a 15% decline in the prevalence of physical inactivity by 2030 [5]. The GAPPA highlights the importance of developing and implementing dedicated comprehensive national policies and strategies to ensure accessible opportunities for active recreation in public spaces such as parks and sports facilities [4]. Namely, GAPPA is focused on leisure-time PA (LTPA). Several large population-based longitudinal follow-up studies demonstrated a non-linear dose-response association between long-term LTPA and all-cause and CVD mortality [6, 7]. Conversely, occupational PA (OPA) in manual workers is associated with an increased risk of CVD and mortality due to maintaining a higher 24-hour heart rate, instantaneously increased blood pressure, insufficient recovery time, lack of worker environmental control, and elevated levels of inflammation [8]. Therefore, PA type (i.e., occupational and leisure time) is one of the important factors in preventing public health problems.

Metabolic syndrome (MetS) is a cluster of cardiometabolic abnormalities, including abdominal obesity, dyslipidemia, hypertriglyceridemia, dysglycemia, and hypertension, leading to cardiovascular disease. Numerous previous studies demonstrated that PA is an independent factor for decreased risk for MetS [9–12]. Especially, a meta-analysis of 17 prospective cohort studies reported that high LTPA is significantly associated with decreased risk for developing MetS (relative risk [95% CI]; vs. low: 0.08 [0.75–0.85] with no difference between moderate LTPA and low LTPA while light domestic PA (housework, yard work, chores) was not associated with MetS [13]. In addition, individuals with at least meet the recommended LTPA (10 MET h/week), 2 times the recommended LTPA (20 MET h/week), and 7 times the recommended LTPA (70 MET h/week) vs. inactive LTPA had 10% (95% CI; 0.86–0.94), 20% (95% CI; 0.74–0.88), and 53% (95% CI; 0.34–0.64) decreased risk for developing MetS. Yet, Holtermann et al. determined that higher OPA vs. lower OPA is an increased risk of CVD (hazard ratio (HR) [95% CI]; higher OPA: 1.16 [1.04–1.29], very higher OPA: 1.35 [1.13.1.61]). In addition, individuals with CVD had an increased risk of developing re-current CVD (HR: 1.15 [0.19–1.45]). Therefore, the association between PA with MetS, cardiometabolic disease, and all-cause mortality may depend on specific-domain PA.

To the best of our knowledge, regular MVPA can positively affect the prevention/treatment of cardiometabolic abnormalities. Nevertheless, the impact of different MVPA domains on MetS risk has yet to be identified among South Koreans. Therefore, the purpose of the study on Korean adults was as follows; (1) to compare the anthropometrics measurement, cardiometabolic decrease risk factor, surrogate markers of insulin resistance, and liver functions in LTPA status, (2) to determine whether LTPA vs. OPA vs. total MVPA is associated with a decreased risk of developing MetS, (3) to identify the dose-response relationship between the LTPA vs. OPA vs. total MVPA with MetS.

## Methods

### Data source

This study utilized data from the 2014 to 2021 Korea National Health and Nutrition Examination Survey (KHANES), which is a nationwide, population-based, cross-sectional study that collects demographic, anthropometric measurements, and health information among South Korean conducted by the Korea Disease Control and Prevention Agency (KCDC). The Institutional Review Board approved the protocol for the KNHANES procedure at the KCDC.

### Participants

The initial data was obtained from 35,874 Korean adult individuals between the ages of 18 and 64 who did not have an under-weight (18.5 kg/m2) in the present study (2014 = 4,451, 2015 = 4,472, 2016 = 4,825, 2017 = 4,912, 2018 = 4,921, 2019 = 5,035, 2020 = 4,487, 2021 = 4,163). The under-weight individuals were excluded from the present study due to avoid potential confounding effects from the differences in pathophysiology between under-weight and obese. Prior to conducting statistical data analyses, a total of 4,316 were excluded for missing or refusing to respond to variables (household income level, education level, occupation, myocardial infarction or angina pectoris, family health history, systolic blood pressure [SBP], height, weight, waist circumference [WC], fasting plasma glucose [FPG], HbA1c, HDL-C and Korean version of the Global Physical Activity Questionnaire (K-GPAQ)). Therefore, the final cohort population included a total of 31,558 individuals.

### Metabolic syndrome

We applied the diagnosis of the MetS based on the criteria of abdominal obesity, increased blood pressure, impaired glucose, hypertriglyceridemia, and low HDL cholesterol suggested by updated modified NCEP-ATP III [14]. Furthermore, given that WC criteria differ from other countries and ethnicities, the South Korean WC cut-off points proposed by the Korean Society for the Study of Obesity (KSSO) were applied to utilize abdominal obesity [15]. Therefore, MetS was defined as having three or more of the following five components; (1) WC > 90 cm in males or > 85 cm in females, (2) FPG ≥ 100 mg/dl, (3) triglyceride (TG) ≥ 150 mg/dl, (4) HDL-C < 40 mg/dl in male or < 50 mg/dl in female, (5) SBP ≥ 130 mmHg and/or DBP ≥ 85 mmHg.

### Physical activity

We assessed LTPA and OPA using the K-GPAQ [16, 17]. The OPA and LTPA were calculated as the sum of the two times the minutes of vigorous activity time per week plus the minutes of moderate activity time per week. The OPA and LTPA status were classified into four categories: (a) 0 min/week, (b) 1 to 149 min/week, (c) 150 to 299 min/week, and d) ≥ 300 min/week [2, 18]. In addition, we calculated total MVPA as a sum of OPA and LTPA and further classified it into six groups; (a) 0 min/week, (b) 1 to 149 min/week, (c) 150 to 299 min/week, (d) 300 to 449 min/week, (e) 450 to 599 min/week, f) ≥ 600 min/week.

### Demographic & physical characteristics

The demographic information included age, sex, smoking status (never smoked, former smoked, smoker), education status (elementary school, middle school, high school, undergraduate), household income level (quartile), occupation (worker, jobless), alcohol consumption (non-alcoholic, alcoholic), and family health history of chronic diseases. Physical characteristics included height (cm), weight (kg), BMI (kg/m2), and WC (cm).

### Cardiometabolic disease risk factors and liver function

Cardiometabolic disease risk factors included SBP (mmHg), DBP (mmHg), mean arterial pressure (MAP; mmHg), TC (mg/dl), HDL-C (mg/dl), TG (mg/dl), FPG (mg/dl), and HbA1c (mg/dl). In addition, we calculated surrogate markers of insulin resistance based on TG, HDL, and glucose, such as TG/HDL-C and TyG (triglyceride-glucose index; ln [FPG (mg/dl)× TG (mg/dl) / 2]) [19, 20].

### Statistical analysis

All data were expressed as the mean (M), standard error of the mean (SEM), frequency (n), and percentage (%). To compare categorical data, including demographic characteristics, and the presence of MetS, we used the chi-square statistic. For comparing continuous variables, including age, LTPA, OPA, anthropometrics, metabolic risk factors, surrogate markers of insulin resistance, and liver function in both the total cohort and sub-cohort population, we conducted one-way ANCOVA, with age and sex as covariates. Logistic regression models were used to estimate the odds ratio (OR) and 95% confidence intervals (CI) between MetS and total MVPA, LTPA, and OPA in the total cohort after adjusting for age, sex, smoking status, household income level, education level, alcohol consumption, and family history of chronic disease. Furthermore, we used restricted cubic spline (RCS) models to investigate the dose-response relationships of MetS with total MVPA, LTPA, and OPA. The RCS models incorporated 3 knots specified at the 10th, 50th, and 90th percentiles, and adjusted for the same confounding factors as used in the logistic regression models. We used the R statistical software package (V. 4.2.2) for all analyses. The significance level was set at 0.05.

## Results

Table 1 shows demographic information, including age, sex, smoking status, education level, household income level, occupation, alcohol consumption status, family health history, and presence of MetS, OPA, and LTPA for participants in this study. The proportion of individuals with a 0 min/week of LTPA, 1 to 149 min/week of LTPA, 150 to 299 min/week of LTPA, and ≥ 300 min/week of LTPA among the total cohort was 67% (n = 21,137), 14% (n = 4,430), 10% (n = 3,037), and 9% (n = 2,954), respectively. Age decreased gradually from 0 min/week of LTPA to 1 to 149 min/week of LTPA to 150 to 299 min/week of LTPA to ≥ 300 min/week of LTPA. In addition, there was a progressive increase in the proportion of females, the highest household income level (Q4), and the presence of non-MetS, from 0 min/week of LTPA to 1 to 149 min/week of LTPA, then to 150 to 299 min/week of LTPA, and finally to ≥ 300 min/week of LTPA.

**Table 1:** Demographic for participants in this study

		Leisure MVPA min/week (N = 31,552)				
	Variables	0 (n = 21,136)	0 to 149 (n = 4,083)	150 to 299 (n = 3,384)	≥ 300 (n = 2,949)	*P*
	Age (years)	44.5 ± 0.1	43.1 ± 0.2	41.9 ± 0.2	41.2 ± 0.2	< 0.001
Sex	Female, n (%)	12,730 (60.2)	2,120 (51.9)	1691 (50.0)	1172 (39.7)	< 0.001
Smoking	Never smoked, n (%)	13,388 (63.3)	2,454 (60.1)	2110 (62.4)	1649 (55.9)	< 0.001
	Former smoker, n (%)	3,045 (14.4)	817 (20.0)	646 (19.1)	609 (20.7)	
	Smoker, n (%)	4,703 (22.3)	812 (19.9)	628 (18.6)	691 (23.4)	
Education	≦ Elementary School, n (%)	1,993 (9.4)	127 (3.1)	84(2.5)	73 (2.5)	< 0.001
	≦ Middle School, n (%)	2,243 (10.6)	200 (4.9)	166 (4.9)	167 (5.7)	
	≦ High School, n (%)	8,431 (39.9)	1,350 (33.1)	1,223 (36.1)	1,180 (40.0)	
	≧ Undergraduate, n (%)	8,469 (40.1)	2,406 (58.9)	1,911 (56.5)	1,529 (51.8)	
House hold income level	Quartile 1, n (%)	2,306 (10.9)	227 (5.6)	182 (5.4)	176 (6.0)	< 0.001
	Quartile 2, n (%)	5,385 (25.5)	762 (18.7)	613 (18.1)	499 (16.9)	
	Quartile 3, n (%)	6,720 (31.8)	1,409 (34.4)	1,058 (31.3)	904 (30.7)	
	Quartile 4, n (%)	6,725 (31.8)	1,685 (41.3)	1,531 (45.2)	1,370 (46.4)	
Occupation	Workers, n (%)	14,553 (68.9)	2,895 (70.9)	2,045 (60.4)	1,941 (65.8)	< 0.001
	Jobless, n (%)	6,583 (31.1)	1,158 (29.1)	1,339 (39.6)	1,008 (34.2)	
Alcohol consumption status	Non-alcoholic, n (%)	13,980 (66.1)	2,655 (65.0)	2,130 (63.2)	1,718 (60.4)	< 0.001
Family health history, n (%)		13,560 (64.2)	2,732 (66.9)	2,209 (65.3)	1,882 (63.8)	< 0.001
Metabolic Syndrome, n (%)		5,116 (24.2)	844 (20.7)	619 (18.3)	483 (16.4)	< 0.001
Occupational MVPA (min/week)		75.2 ± 2.2	89.3 ± 4.9	105.0 ± 6.1	157.6 ± 8.4	< 0.001
Leisure-time MVPA (min/week)		0.0 ± 0.0	84.3 ± 0.6	227.3 ± 0.8	638.5 ± 22.9	< 0.001

cardiovascular disease. MVAP; moderate to vigorous physical activity

Table 2 shows anthropometric measurements, blood pressure, metabolic characteristics, surrogate markers of insulin resistance, and liver function in the total cohort population. WC was higher in 0 min/week of LTPA compared with other groups, including the 1 to 149 min/week of LTPA, 150 to 299 min/week of LTPA, and a ≥ 300 min/week of LTPA. In addition, a ≥ 300 min/week of LTPA was lowest in DBP than a 0 min/week of LTPA and a 1 to 149 min/week of LTPA, with no difference between a ≥ 300 min/week of LTPA and a 150 to 299 min/week of LTPA. Despite a lower BMI and SBP, in the 1 to 149 min/week of LTPA compared to ≥ 300 min/week of LTPA, there was progressive worse in HDL-C, TG, and TyG. In addition, FPG and TG/HDL-C gradually decreased from 0 min/week of LTPA to 1 to 149 min/week of LTPA to 150 to 299 min/week of LTPA and a ≥ 300 min/week of LTPA. HbA1c was higher in the 0 min/week of LTPA compared with a 1 to 149 min/week of LTPA, a 150 to 299 min/week of LTPA, and a ≥ 300 min/week of LTPA. AST was higher in a ≥ 300 min/week of LTPA compared with a 0 min/week of LTPA, a 1 to 149 min/week of LTPA, and a 1 to 149 min/week of LTPA.

**Table 2:** Anthropometric and cardiometabolic disease risk factors according to PA status in the total cohort population

	Leisure MVPA min/week (N = 31,552)				
Variables	0 min/week (n = 21,137)	0 to 149 min/week (n = 4,430)	150 to 299 min/week (n = 3,037)	≥ 300 min/week (n = 2,954)	*ANCOVA P* value
**Anthropometrics**					
BMI (kg/m^2^)	23.9 ± 0.0	23.7 ± 0.1	23.9 ± 0.1	24.0 ± 0.0	< 0.01
WC (cm)	82.2 ± 0.1 a	81.4 ± 0.1 a	81.2 ± 0.2 a	81.0 ± 1.7 b	< 0.001
**Blood pressure**					
SBP (mmHg)	115.9 ± 0.1 a	114.5 ± 0.2 b	115.1 ± 0.2 ab	115.6 ± 0.3 a	< 0.001
DBP (mmHg)	76.1 ± 0.1 b	75.8 ± 0.1 ab	75.9 ± 0.2 ab	75.4 ± 0.2 a	< 0.01
**Metabolic risk**					
TC (mg/dl)	193.2 ± 0.3	194.3 ± 0.5	193.0 ± 0.7	193.6 ± 0.7	0.308
HDL-C (mg/dl)	51.9 ± 0.1 a	53.1 ± 0.2 b	53.7 ± 0.2 c	54.8 ± 0.2 d	< 0.001
TG (mg/dl)	137.6 ± 0.8 a	128.9 ± 1.7 b	121.0 ± 2.0 c	120.2 ± 2.0 d	< 0.001
FPG (mg/dl)	99.7 ± 0.1 a	98.0 ± 0.3 b	96.9 ± 0.4 c	96.8 ± 0.4 c	< 0.001
**Insulin resistance markers**					
TG/HDL-C	3.09 ± 0.02 a	2.82 ± 0.05 b	2.63 ± 0.06 c	2.56 ± 0.06 c	< 0.001
TyG	8.60 ± 0.00 a	8.54 ± 0.01 b	8.49 ± 0.01 c	8.44 ± 0.01 d	< 0.001

*Note*: ANCOVA; adjusted for age and sex. The same alphabet is not significantly different groups (a, b, c)

**Fig. 1:**
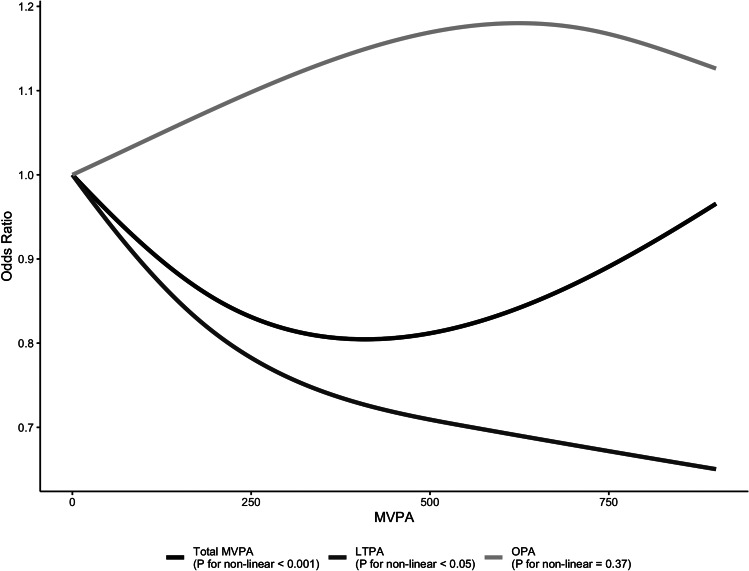
Dose–response relationship of domain-specfic moderate-vigorous physical activity (MVPA) with metabolic syndrome (total MVPA vs. leisure tiem MVPA [LTPA] vs. occupational MVPA [OPA])

Multinomial logistic regression analyses were conducted to identify the odds ratios and 95% CI for MetS by the LTPA status (Table 3). The MetS in individuals with a 1 to 149 min/week of LTPA, a 150 to 299 min/week of LTPA, and a ≥ 300 min/week of LTPA among Korean adults were 0.860-, 0.770-, and 0.616 times decreased risk compared with the 0 min/week of LTPA. However, there was no significant association between PA and MetS. In addition, there is a significant non-linear association between the total MVPA and LTPA with the risk of MetS while no non-linear relationship between OPA and risk of MetS was founded (Fig. 1).

## Discussion

The present study identified the impact of different types of PA (total MVPA vs. LTPA vs. OPA) on the risk of developing MetS among Korean adults. Our study revealed; (1) a ≥ 300 min/week of LTPA and a 150 to 299 min/week of LTPA showed better outcomes in metabolic characteristics and surrogate markers of insulin resistance compared with 0 min/week of LTPA regardless of adiposity status; (2) risk of MetS in ≥ 300 min/week of LTPA was lower than in a 0 min/week, 1 to 149 min/week, and 150 to 299 min/week; (3) total MVPA and LTPA were significantly associated with a risk of the MetS in a curvilinear dose-response curve; (4) no significant effects of a non-linear relationship between OPA and risk of the MetS.

All domain physical activity cannot positively affect health outcomes. Especially, PA of manual work does not contribute to health promotion as LTPA [21]. Our findings show a dose-response relationship between LTPA and the risk of metabolic syndrome, whereas no association was found between OPA and the risk of metabolic syndrome. In addition, individuals with ≥ 300 min/week of LTPA were associated with a decreased risk of developing MetS (− 38.4%) compared individuals with 0 min/week of LTPA. Of note, in the joint analysis, the ≥ 300 min/week of LTPA was strongly associated with decreased risk for MetS (95% CI; 0.553–0.689) compared with 0 min/week-, 1 to 149 min/week- (95% CI; 0.788–0.938), and 150 to 299 min/week of total MVPA (95% CI; 0.698–0.849) because there was no AUC 95% CI overlap between ≥ 300 min/week of LTPA and other groups. A meta-analysis of prospective cohort studies showed that a high level of LTPA is strongly associated with decreased risk of developing MetS (relative risk (RR) [95% CI], vs. low level of LTPA; 0.80 [0.75–0.85]) compared moderate level of LTPA (vs. low level of LTPA; 0.95 [0.91-1.00] – supports our results [13]. Furthermore, the 450 to 559 min/week of total MVPA was strongly associated with decreased risk for MetS (95% CI; 0.513–0.732) compared with 0 min/week-, 1 to 149 min/week- (95% CI; 0.782–0.934), and 150 to 299 min/week of total MVPA (95% CI; 0.760–0.933), with no difference between ≥ 600 min/week-, 300 to 449 min/week-, and 150 to 299 min/week of total MVPA. We speculated that the optimal range of total MVPA and LTPA for decreasing the risk of MetS are 450 to 559 min/week and ≥ 300 min/week, respectively. Although we showed no associations between OPA and risk of MetS among Korean adults, higher OPA is associated with physical deterioration and functional impairments caused by heavy physical labor [22]. While OPA may not directly impact the risk of MetS, it is evident that engaging in high occupational workload can have detrimental effects on individuals’ health such as musculoskeletal disorders, chronic pain, and decreased functional movement [23]. Notably, several studies suggested that repetitive bouts of resistance physical tasks without adequate recovery time could induce cardiovascular morbidity and mortality [8, 24]. Therefore, a future study is warranted to examine the association of specific occupation types with the risk of MetS in Korean adults.

Our study findings are in line with accordance with previous studies supporting the interaction between LTPA and the socio-demographics [25]. In the present study, the difference in the proportion of sex, education level, and household income level may support meeting 2025 WHO global PA target (a 10% decrease in inactive in all countries) [26]. As the level of LTPA gradually decreased, the age, and proportion of females, progressed to increase, and individuals with the highest education level (undergraduate) and household income level (Q4) had greater participation in LTPA (≥ 300 min/week). In addition, the prevalence of MetS decreased gradually from individuals with inactive LTPA to those engaging in 1 to 149 min/week, 150 to 299 min/week, and last to those with ≥ 300 min/week of LTPA. Strain et al. demonstrated levels of specific-domain PA among 327,789 adults from 104 countries using GPAQ from 2002 to 2019 [27]. This study supported our findings that the contribution of MVPA through LTPA increased gradually from low- (4%) to lower-middle- (8%) to upper-middle (13%) to high-income countries (28%) and the prevalence of physical inactivity is higher in female (27%) compare with male (20%) among 142 counties [28]. Furthermore, previous studies have shown that those with higher levels of education status lead to earned income more and engage with their healthier behavior (PA), resulting in the prevention/modification of potential health risks [29, 30]. Our study provides evidence to public health policy to promote LTPA habits among individuals socially disadvantaged, suggesting that females and low levels of household income and education should be considered when establishing implications for planning policy.

The present study findings align with previous literature supporting the PA guideline for health outcomes. Additionally, our study results suggest that LTPA and WC are strongly associated with metabolic disease risk and surrogate markers of insulin resistance regardless of BMI. In addition, although there are non-linear pattern results between LTPA and blood pressure variables, the prevalence of hypertension (SBP ≥ 140 or DBP ≥ 90) was highest in 0 min/week of LTPA (13.8%), followed by 1 to 149 min/week of LTPA (12.6%), a 150 to 299 min/week of LTPA (12.6%), and a ≥ 300 min/week of LTPA (12.3%). Crichton & Alkerwi demonstrated that individuals who participated 0.5 to 1 h/day for vigorous PA have greater HDL-C compared with those who were less than status (< 0.5 h/day), and TG gradually decreased in linear trends with increasing vigorous PA time. Additionally, previous studies reported that physical inactivity is the main independent risk factor for insulin resistance [31] and increasing and/or maintaining PA was associated with decreased risk for insulin resistance [32]. Therefore, LTPA is a potentially modifiable factor associated with cardiometabolic disease risk factors.

The strength of our study are as follows; (1) first-time comprehensive examination of the relationship between the PA domain and the risk of MetS in a large population-based dataset in South Korea; (2) using a non-linear mixed effects statistical modeling approach; (3) utilization of rigorous controlled potential confounders. Nevertheless, our study had some limitations that should be noted when interpreting the results. A limitation of the present study is the cross-sectional design which does not allow any cause-effect relationship. Further, we only examined the relationship between specific MVPA intensity and the risk of MetS. Therefore, further longitudinal studies are needed to identify the association between various PA types, i.e., sedentary and light PA, and cardiometabolic abnormalities.

In conclusion, our findings demonstrated that higher levels of LTPA are strongly associated with a lower risk of MetS with a dose-response curve, and LTPA was significantly associated with the risk of MetS compared with OPA in South Korea. Therefore, the MVPA domain is an independent factor of the risk of MetS.

**Table 3:** The odds ratio for MetSyn risk by physical activity domain

Variables		Group	*B*	S. E	Odds ratio	95% CI					
											LTPA
MetSyn		0 min/week			1.000						
		0 to 149 min/week	-0.151	0.044	0.860*	0.788–0.938					
		150 to 299 min/week	-0.262	0.050	0.770*	0.698–0.849					
		≥ 300 min/week	-0.481	0.055	0.618*	0.553–0.689					
OPA											
MetSyn		0 min/week			1.000						
		0 to 149 min/week	0.062	0.335	1.064	0.551–2.054					
		150 to 299 min/week	0.059	0.133	1.061	0.817–1.377					
		≥ 300 min/week	-0.067	0.052	0.935	0.844–1.036					
Total MVPA											
		0 min/week			1.000						
MetSyn		0 to 149 min/week	-0.157	0.045	0.855*	0.782–0.934					
		150 to 299 min/week	-0.172	0.052	0.842*	0.760–0.933					
		300 to 449 min/week	-0.315	0.075	0.730*	0.630–0.845					
		450 to 599 min/week	-0.490	0.091	0.613*	0.513–0.732					
		≥ 600 min/week	-0.274	0.053	0.760*	0.685–0.843					

*Note*: adjusted for age, sex, smoking status, household income level, education level, occupation, alcohol consumption, family hallah history of chronic disease; *P < .001

## Data Availability

The datasets generated analyzed during the current study are available in the 2014 to 2021 KHANES (https://knhanes.kdca.go.kr/knhanes/sub04/. sub04_04_01.do)
